# Vitamin K Deficiency Induced by Warfarin Is Associated With Cognitive and Behavioral Perturbations, and Alterations in Brain Sphingolipids in Rats

**DOI:** 10.3389/fnagi.2018.00213

**Published:** 2018-07-16

**Authors:** Sahar Tamadon-Nejad, Bouchra Ouliass, Joseph Rochford, Guylaine Ferland

**Affiliations:** ^1^Center for Addiction and Mental Health, Toronto, ON, Canada; ^2^Montreal Heart Institute Research Centre, Montreal, QC, Canada; ^3^Douglas Institute Research Center, Montreal, QC, Canada; ^4^Department of Psychiatry, McGill University, Montreal, QC, Canada; ^5^Département de Nutrition, Université de Montréal, Montreal, QC, Canada

**Keywords:** vitamin K, menaquinone-4, nutritional deficiency, brain, cognition, sphingolipids, rat model

## Abstract

Initially discovered for its role in blood coagulation, there is now convincing evidence that vitamin K (VK) has important actions in the nervous system. In brain, VK is present in the form of menaquinone-4 (MK-4), a byproduct of the main dietary source, phylloquinone. It contributes to the biological activation of various proteins (i.e., Gas6), and participates in the synthesis of sphingolipids, a class of lipids widely present in brain cell membranes with important cell signaling functions. In a previous study, we reported that lifetime consumption of a low VK diet resulted in mild cognitive impairment in aged rats, a finding associated with an alteration of the sphingolipid profile. To confirm the role of VK as it relates to sphingolipids, cognition, and behavior outside the context of aging, we conducted a study of acute VK deficiency using a pharmacological model of VK deficiency in brain. In this procedure, rats (8 weeks) are maintained on a ratio of warfarin (a VK antagonist) to VK whereby coagulation is maintained while inducing VK deficiency in extrahepatic tissues. After 10 weeks of treatment, rats who were subjected to the warfarin plus phylloquinone protocol (WVK) exhibited longer latencies in the Morris water maze test as well as lower locomotor activity and exploratory behavior in the open field test, when compared to control rats. The WVK treatment resulted in a dramatic decrease in MK-4 level in all brain regions despite the presence of high local concentrations of phylloquinone, which suggests an inhibition of the biosynthetic MK-4 pathway in the presence of warfarin. Additionally, WVK treatment affected sphingolipid concentrations in key brain regions, notably those of the ganglioside family. Finally, brain MK-4 was correlated with performances in the open field test. This study confirms the modulatory role of VK in cognition and behavior and the implication of sphingolipids, notably those of the ganglioside family.

## Introduction

The preservation of cognitive abilities and mobility is of primary importance to older people as cognitive decline and physical frailty result in loss of independence and decreased quality of life. Lifestyle factors, notably nutrition, are increasingly being confirmed as powerful modulators of cognition and physical functions in older age (Landi et al., 2015; Tucker, 2016; Vauzour et al., 2017). Historically discovered for its role in blood coagulation, vitamin K (VK) has recently emerged as an important nutrient for brain function. Vitamin K occurs naturally in two forms, phylloquinone which originates from plants, is the main dietary source, while the menaquinones, which are of bacterial origin, constitute a family of compounds with unsaturated isoprenyl side chains of various lengths (Ferland, 2012a; Shearer and Newman, 2014). One of the menaquinones, menaquinone-4 (MK-4), is not a common product of bacterial synthesis but is synthesized from phylloquinone (Nakagawa et al., 2010). Under normal conditions, VK in brain is overwhelmingly in the form of MK-4 (Thijssen and Drittij-Reijnders, 1994) where it has been shown to represent more than 98% of total VK in brains of Sprague-Dawley rats (Carrié et al., 2004, 2011).

**FIGURE 1:**
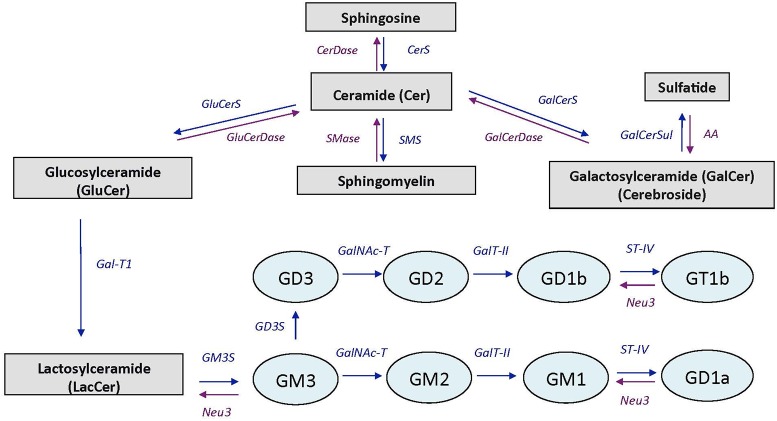
Simplified scheme of sphingolipid and ganglioside pathways. CerDase, ceramidase; CerS, ceramide synthase; GluCerS, glucosylceramide synthase; GluCerDase, glucosylceramidase; SMase, shingomyelinase; SMS, sphingomyelin synthase; GalCerDase, galactosylceramidase; GalCerS, galactosylceramide synthase; GalCerSul, galactoceramide sulfotransferase; AA, arylsufatase A; Gal-T1, -β-1,3-galactosyltransferase; GM3S, GM3 synthase (ST3 β-galactoside-α-2,3-sialyltransferase-5); GD3S, GD3 synthase (ST8 α-*N*-acetyl-neuraminide-α-2-8-sialyltransferase); GalNAc-T, β-1-4-*N*-acetyl- galactosaminyltransferase-1; GalT-II, galactosyltransferase; ST-IV, ST6 (α-*N-*acetyl-neuraminyl-2,3-β-galactosyl-1,3)-*N-*acetylgalactosaminide-α-2,6-siasyltransferase; Neu3, sialidase 3. Adapted from Giussani et al. (2014).

In brain, VK is involved in sphingolipid metabolism, a group of complex lipids highly enriched in the nervous system where they are major components of cell membranes. Major sphingolipids in the central nervous system include ceramides, sphingomyelin, cerebrosides, sulfatides, and gangliosides (Olsen and Færgeman, 2017). Ceramide constitutes the basal building block for the more complex sphingolipids which are generated by attaching various head groups in the C1 position of ceramide. Sphingomyelin, cerebrosides, and sulfatides are particularly present in oligodendrocytes and myelin (white matter), whereas gangliosides are major components of neuronal membranes (gray matter; Posse de Chaves and Sipione, 2010). Ganglioside biosynthesis occurs by sequential glycosylation reactions *via* two major pathways, designated the “a-pathway” and the “b-pathway” (Giussani et al., 2014; Schnaar, 2016). A simplified scheme of sphingolipid and ganglioside pathways is presented in **Figure 1**.

Initially appreciated for their structural role, sphingolipids are now viewed as key players of important cellular events such as neuronal cell proliferation, differentiation and senescence, synaptic transmission, neuronal–glial interaction, and myelin stability (Bartke and Hannun, 2009; Schengrund, 2015; Olsen and Færgeman, 2017). Furthermore, research conducted in recent years have linked alterations in sphingolipid metabolism to the aging process (Cutler et al., 2004; Ledesma et al., 2012), neurodegenerative disorders such as Alzheimer and Parkinson diseases (Han, 2010; van Echten-Deckert and Walter, 2012; Ariga, 2014; Gualtierotti et al., 2017), psychiatric disorders, and emotional behaviors (Schneider et al., 2017). Ceramides generated by the hydrolysis of sphingomyelin by the sphingomyelinase enzymes, have been shown to accumulate during aging (Cutler et al., 2004; Costantini et al., 2005; Babenko and Semenova, 2010) and to be increased by up to threefolds in the brains of Alzheimer’s disease patients when compared with age-matched controls (Han et al., 2002; Cutler et al., 2004). Work conducted in recent years have also provided strong evidence for an implication of the ceramide–sphingomyelin pathway in behavioral extinction (Huston et al., 2016), and in emotional behaviors and psychiatric disorders (Schneider et al., 2017). In a series of studies, the acid sphingomyelinase–ceramide pathway was shown to play an important role in genetically (Kornhuber et al., 2014; Müller et al., 2017) and stress-induced depression (Gulbins et al., 2013; Oliveira et al., 2016).

**FIGURE 2:**
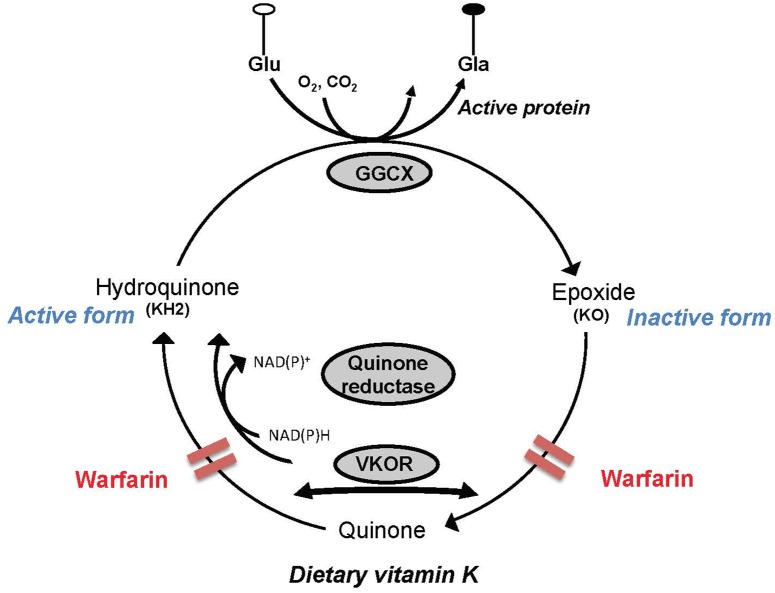
Simplified depiction of the vitamin K (VK) cycle. VK acts as a cofactor for the γ-glutamyl carboxylase enzyme (GGCX) involved in the posttranslational synthesis of gamma-carboxyglutamic acid (Gla) from glutamic acid (Glu) residues contained in VK precursor proteins. In the course of the catalytic sequence, hydroquinone (active form) is oxidized to VK 2,3-epoxide (KO), which in turn is recycled to the quinone and hydroquinone forms by the VK oxidoreductase (VKOR). Activity of VKOR is inhibited by 4-hydroxycoumarin derivatives such as warfarin. In the liver where coagulation factors are produced, a coumarin insensitive NAD(P)H-dependent quinone reductase enzyme operates at high tissue concentrations of VK and can therefore support carboxylation of the hepatic blood coagulation factors in the presence of coumarins.

In addition to their implication in sphingolipid metabolism, the K vitamers act as cofactors in a carboxylation reaction that results in the posttranslational modification of the glutamic acids contained in precursor proteins, the best known of which are those involved in hemostasis (Suttie, 2009). However, the VK-dependent proteins Gas6 and protein S are present in brain where they are known to possess cell signaling actions in neurons and the glia, and antithrombotic activity (reviewed in Ferland, 2012b). Activation of the VK-dependent proteins involves a series of reactions where the VK oxidoreductase enzyme supports the recycling of the K vitamers. In extrahepatic tissues including brain, this enzyme is inhibited by 4-hydroxycoumarin derivatives, such as warfarin. However, in liver where coagulation factors are produced, a coumarin insensitive NAD(P)H-dependent quinone reductase enzyme operates at high tissue concentrations of VK. By maintaining a specific ratio of warfarin to phylloquinone, it is hence possible to induce VK deficiencies in extrahepatic tissues while maintaining the hemostatic functions (Wallin et al., 1978; Tie and Stafford, 2016; **Figure 2**).

In a previous study, we reported that lifetime consumption of a low VK diet results in mild cognitive impairment in aged rats (i.e., 20 months), a finding associated with an alteration of the sphingolipid profile (Carrié et al., 2011). Vitamin K status is influenced by age (Huber et al., 1999; Ferland et al., 2016a) and sphingolipid metabolism is altered during aging. In light of this and to confirm the role of VK as it relates to sphingolipids, cognition, and behavior outside the context of aging, we conducted a study of acute VK deficiency by subjecting rats to the WVK protocol.

## Materials and Methods

### Animals

All experimental procedures were approved by the Animal Care Committee of the Université de Montréal according to the guidelines of the Canadian Council on Animal Care. Male Wistar rats (age 8 weeks; initial body weights 175–250 g) were obtained from Charles Rivers, Canada. Rats were housed two per cage, in a room maintained at 22°C with a 12-h light/dark cycle. Rats were kept in the same housing conditions and rat facility throughout the experimental period. Rats had free access to water and food.

### Warfarin-Induced Vitamin K Deficiency Protocol

The warfarin-induced VK deficiency protocol used in the present study was that developed by Price et al. (1982) as modified by Essalihi et al. (2003). Specifically, rats from the experimental group (WVK) were treated with 14 mg/kg/day W in their drinking water and subcutaneous phylloquinone (85 mg/kg/day) injections, three times per week for 10 successive weeks. Phylloquinone injections were started 1 week before warfarin administration and warfarin dosing was adjusted three times per week by checking the drunken volume. Control (C) rats were treated with normal water and injected with saline three times per week for 10 successive weeks. Both groups were fed an AIN-93-based diet containing 750-μg phylloquinone/kg diet. The health of the rats was monitored daily throughout the experimental period and included assessment of general appearance and behavior, i.e., quality of fur/grooming, presence of epistaxis or other bleeding, posture, mobility in the cage, response to external stimuli, etc. Food intake and body weights were recorded once and twice per week, respectively. Blood clotting capacity, i.e., prothrombin time of animals was monitored once a week using blood samples from the tail vein using a Coagucheck device (Roche, Canada).

### Behavioral Testing

At the end of the treatment period, rats from both groups were subjected to the Morris water maze (Morris, 1984), the open field (Kelley, 1993), and the elevated plus maze (Pellow et al., 1985), these tests assessing spatial learning and memory, locomotor activity and exploration, and anxiety, respectively.

#### Morris Water Maze

Morris water maze test consisted of a large, circular, metal pool (diameter: 150 cm) filled approximately half-way (30 cm) with 22°C water. A fixed invisible platform (10 cm^2^) which was submerged below the water surface (∼2 cm) was placed in the center of one of the four quadrants of the pool. Several objects or images (e.g., circles, squares, and triangles) were hung on the walls of the room in which the test was conducted, so the rats could use them as visual stimuli for navigating in the maze. Each day of the trial, rats were released in the water in one of the four quadrants randomly. Testing was conducted daily over five consecutive days, each rat being given three trials per day with an inter-trial interval of 20 min. Once the rat located the platform, it was allowed to remain on it for ∼30 s. If a rat did not find the platform after 120 s of swimming, it was gently put on it by the experimenter. Learning performance was based on the mean of three daily trials.

##### Probe trial

On day 6, memory retention was further assessed by removing the platform from the pool and allowing the rats to swim freely during 30 s. The pool was divided into the same four quadrants as for the learning condition and the percent time spent in each quadrant was computed. Rats were allowed two trials.

##### Cue test

Immediately after the probe trial, a cue test was conducted to ensure that poor performances were not due to visual deficits. In this test, rats had to find the platform that had been rendered visible by lowering the pool water level (2 cm below the top of the platform). Rats were allowed two trials. Latencies to find the platform, swim speed, and time in each quadrant were monitored with a camera mounted above the pool and recorded with a DVD recorder. Latencies to find the platform, swim speed and time spent in each quadrant were analyzed using the TopScan 2.0 system (Clever Systems Inc., Reston, VA, United States).

#### Open Field

The open field consisted of a black wooden arena measuring 60 cm × 50 cm × 50 cm with the floor divided in 25 cm × 10 cm squares. After 30 min of adaptation to the room, rats were placed individually into the open field and allowed to explore it freely for 5 min. Locomotor activity was based on total distance moved (cm) and total number of square crossed, while exploratory behavior was assessed by the animals’ % time spent in center squares and number of center crossings. Motor activity and exploratory components were recorded using a HVS 2020 tracker over 1 day, and analyzed by the Field 2020’s software (HVS Image).

#### Elevated Plus Maze

Anxiety-like behavior was measured using the elevated plus maze, a test that relies on the rodent’s innate fear of open spaces and height. The elevated plus maze consisted of a gray wooden cross with four arms (90 cm × 8 cm) that was elevated 70 cm from the floor. Two opposite arms were open, while the other two were enclosed by side end walls (10-cm high). After 30 min of adaptation to the room, rats were placed in the middle of the intersection of the four arms facing an open arm and their behavior was recorded for 5 min. During this period, the total time spent in the open arms, number of open arm entries, and the total number of arm entries were measured. The test was conducted once.

### Biochemical Analyses

After completion of the behavioral tests, rats were anesthetized with pentobarbital and bled from the abdominal aorta. The brains were gently removed on ice and dissected into midbrain, prefrontal cortex, hippocampus, striatum, and sensorimotor cortex. The brain regions were frozen in liquid nitrogen and stored at -80°C until assessments.

#### Vitamin K Analysis

Phylloquinone and MK-4 were quantified by reverse-phase HPLC as previously described (Carrié et al., 2011). Briefly, tissue samples were pulverized in anhydrous Na_2_SO_4_ and extracted with acetone containing an internal standard [2-methyl-3-(3,7,11,15,19-pentamethyl-2-eicosenyl)-1,4 naphthalenedione; GL Synthesis, Inc.]. Dried extracts were then reextracted with a mixture of hexane and water before being further purified by solid phase extraction on silica gel columns (J. T. Baker). Quantitative analysis of phylloquinone and MK-4 was performed by reverse-phase HPLC using a C-18 reverse phase column and fluorescence detection. The calibration standard consisted of a mixture of phylloquinone, MK-4, and 2-methyl-3-(3,7,11,15,19-pentamethyl-2-eicosenyl)-1,4-naphthalenedione at 2 ng in 50 μl. The percent recovery for the samples was calculated from the internal standard and found to be 85–90%.

#### Sphingolipid Analyses

Sphingolipids which included ceramides, sphingomyelin, cerebrosides, sulfatides, and gangliosides, were assessed in the various brain regions as described previously (Carrié et al., 2011). Briefly, lipids were extracted from the brain regions using chloroform:methanol (2:1, v:v) and partitioned according to the method of Folch et al. (1957). Gangliosides were eluted according to the method of Williams and McCluer (1980), and were measured by quantification of free sialic acids according to Jourdian et al. (1971). Ceramides, cerebrosides, sulfatides, and sphingomyelin were loaded onto LC-NH2 columns (Supelco) and eluted sequentially. The sulfatide fraction was further applied to a C-18 silica column. Each fraction was evaporated and suspended in chloroform:methanol (2:1, v:v). Ceramides, cerebrosides, and sphingomyelin were quantified by determination of sphingosine with fluorescamine according to the method of Naoi et al. (1974) and sulfatides with azure A according to the method of Kean (1968).

#### Ganglioside Analyses

Gangliosides subtypes were analyzed by high-performance thin-layer chromatography (HPTLC) using 20 cm × 10 cm silica gel 60 HPTLC plates (Merck, Darmstadt, Germany). Purified gangliosides standards (GT1b, GD1a, GM1, and GD1b) were purchased from Matreya LLC. Each ganglioside standard mixtures were spotted in duplicate on each plate. The HPTLC plates were prewashed with chloroform to eliminate contamination that could affect gangliosides mobility (Ravindranath et al., 2004). After a brief drying period, plates were placed in a chamber containing 200 ml of developing solvent [chloroform/methanol/0.25% aqueous CaCl_2_ (60/35/7.5 v/v/v); Ardail et al., 2003] which had equilibrated for at least 2 h (Ledeen and Yu, 1982). Plates were run for 60 min, air dried, and sprayed with Resorcinol reagent (10 ml of 2% resorcinol in water, 40 ml concentrated HCl and 0.250 ml of 0.1-M copper sulfate; Svennerholm, 1957). Plates were then placed face down on a clean glass cover plate in an oven at 120°C until appearance of the bands (about 20 min; Hauser et al., 2004). The percent distribution of the individual gangliosides was determined by scanning the HPTLC plates and computing the bands using Image J software (version 1.42, National Institutes of Health, Bethesda, MD, United States). Individual bands from each lane were identified by comparison with a standard mixture.

### Statistical Analyses

Statistical analyses were performed using GraphPad Prism (version 6.01). All data were expressed as means ± SEM. Body weight and Morris water maze test were assessed using a mixed, between-within ANOVA model, with WVK treatment as the between-subject effect and time as the repeated measure. Phylloquinone, MK-4, total VK, MK-4/total VK, and sphingolipids were assessed for regional differences by one-way ANOVA followed by Bonferroni or Tukey’s *post hoc* tests. Group difference (C vs. WVK) in Probe trial, Cue test, swimming speed, latencies on individual days of the Morris water maze, open field, and elevated plus maze; prothrombin time; phylloquinone, MK-4, MK-4/total VK, sphingolipids, and ganglioside subtypes within a brain region, were analyzed by Student’s *t*-test. Pearson’s correlation test was performed to estimate the linear relationship between MK-4 and behavioral performances. *p*-Values <0.05 were considered to be statistically significant.

## Results

### Body Weight and Health of Animals

Body weights increased in both C and WVK groups during the 10-week experimental period although the WVK-treated rats gained significantly less weight compared to controls [*F*_(1,200)_ = 102.0; *p* < 0.0001]. Specifically, mean weights of WVK rats were ∼10% [(C) 418.5 ± 5.9 g vs. (WVK) 380.4 ± 7.1 g] and ∼14% [(C) 556.1 ± 12.0 g vs. (WVK) 477.5 ± 12.4 g] lower at weeks 5 and 10 of treatment, respectively (*p* < 0.05). These differences between C and WVK groups are comparable to those reported by other teams having used this model (Price et al., 1982; Essalihi et al., 2003) and are likely due to difference in food intake. Mean food intake for the entire study was 9% lower for the WVK (21.84 ± 0.41 g/day) compared to the control group (24.06 ± 0.49 g/day; *p* < 0.05). General appearance of animals (i.e., grooming) and behavior (i.e., mobility, response to external reflex, etc.) remained normal throughout the study. Importantly, no animal showed signs of bleeding although mean prothrombin times for the study were slightly higher in the WVK than C group [(C) 1.1 s vs. (WVK) 2.6 s; *p* < 0.01].

### Behavioral Testing

#### Morris Water Maze

**FIGURE 3:**
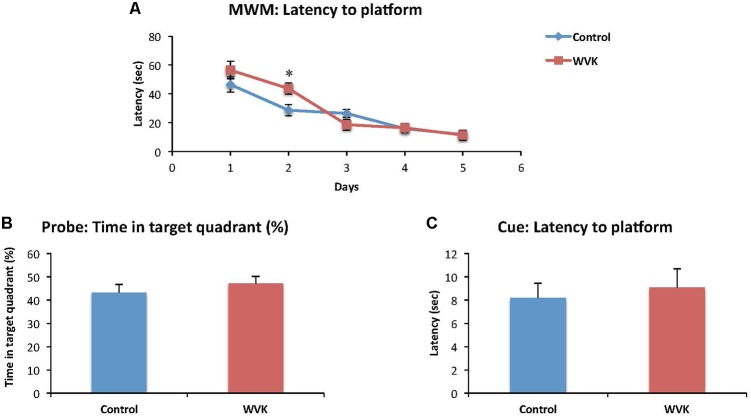
Effect of WVK treatment on spatial learning, memory retention, and cue test in the Morris water maze. Figures show **(A)** the mean latency (s) to reach the submerged platform over the 5-day test; **(B)** the percentage (%) of time spent in the target quadrant with platform removed; **(C)** the mean latency (s) to reach the visible platform. Values are mean ± SEM, *n* = 8–13 rats per group. Data were analyzed by **(A)** repeated-measures ANOVA, followed by Bonferroni *post hoc* test or **(B,C)** Student’s unpaired *t*-test. Statistically different between C and WVK groups, ^∗^*p* < 0.05.

Time to find the hidden platform decreased across the successive training days in both groups [*F*_(4,82)_ = 34.6; *p* < 0.0001], suggesting that learning occurred across trials (**Figure 3A**). However, there was a significant interaction effect suggesting that learning across days differed between groups [*F*_(4,82)_ = 2.7; *p* < 0.05]. Specifically, rats from the WVK group presented significantly longer latencies compared to those of C group on day 2 (*p* < 0.05). Probe trial indicated that both groups had a preference for the quadrant in which the platform was located during the learning trials but the percent time spent in the target quadrant was not statistically different between groups (*p* = 0.45; **Figure 3B**). Finally, performance on the Cue test (*p* = 0.46) and animals’ swim speed (*p* = 0.62) were similar confirming that visual acuity and mobility in the pool were not affected by the WVK treatment (**Figure 3C**).

#### Open Field

**FIGURE 4:**
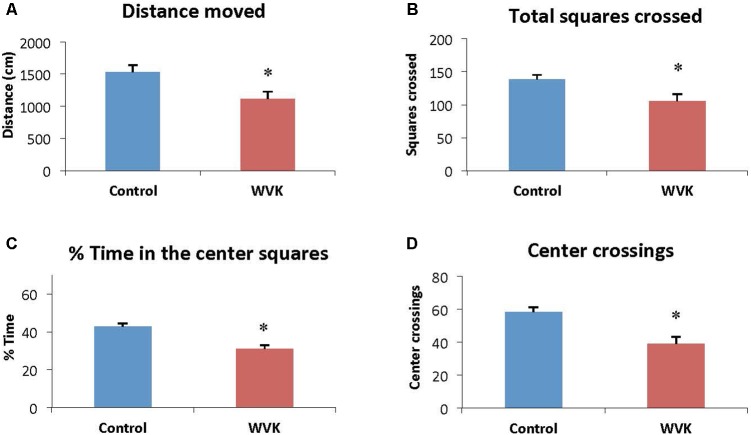
Effect of WVK treatment on locomotor activity and exploratory behavior in the open field. Figures show **(A)** the total distance moved; **(B)** the total number of squares crossed; **(C)** the mean % time spent in center squares; **(D)** the total number of center crossings. Values are mean ± SEM, *n* = 8–13 rats per group. Data were analyzed by Student’s unpaired *t*-test. Statistically different between C and WVK groups, ^∗^*p* < 0.05.

Locomotor activity and exploratory behavior were affected in the experimental group. Specifically, distance moved [*F*_(12,7)_ = 1.26; *t*_(19)_ = 2.67, *p* = 0.015] and total number of squares crossed [*F*_(12,7)_ = 1.42; *t*_(19)_ = 2.76, *p* = 0.012] were significantly reduced compared to control rats (**Figures 4A,B**). Similarly, WVK rats presented lower exploratory behavior compared to their control counterparts with lower percent time spent in the center squares [*F*_(12,7)_ = 1.58; *t*_(19)_ = 4.69, *p* = 0.002] and number of center square crossings [*F*_(12,7)_ = 1.33; *t*_(19)_ = 3.87, *p* = 0.001; **Figures 4C,D**].

#### Elevated Plus Maze

Anxiety as assessed with this paradigm was not affected by the WVK treatment. Time spent in the open arms [*F*_(12,7)_ = 1.31; *t*_(19)_ = 0.94, *p* = 0.35], percent time spent in open arms/total time [*F*_(12,7)_ = 1.34; *t*_(19)_ = 0.55, *p* = 0.59], and percent open arm entries/total entries [*F*_(12,7)_ = 0.27; *t*_(19)_ = 1.33, *p* = 0.20] did not differ significantly between groups (data no shown).

### Vitamin K Status

**FIGURE 5:**
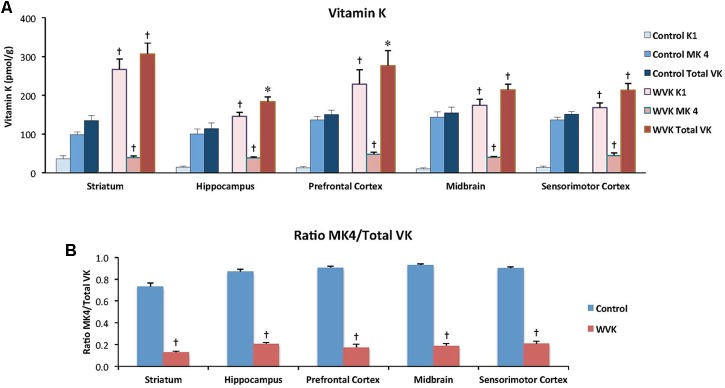
Effect of WVK treatment on VK status in different brain regions in C and WVK groups. Figures show **(A)** phylloquinone (K_1_), menaquinone-4 (MK-4), and total VK concentration in striatum, hippocampus, prefrontal cortex, midbrain, and sensorimotor cortex; **(B)** the ratio of MK-4/total VK in these brain regions. Values are mean ± SEM, *n* = 6–7 rats per group. Differences between brain regions were analyzed for individual vitamer within C and WVK groups by one-way ANOVA. [Control gp. K_1_: *F*_(4,23)_ = 5.687, *p* < 0.002, MK-4: *F*_(4,23)_ = 4.45, *p* < 0.01, total VK: *F*_(4,23)_ = 1.81, *p* = 0.16, MK-4/total VK: *F*_(4,23)_ = 11.90, *p* < 0.001; WVK gp. K_1_: *F*_(4,34)_ = 4.77, *p* < 0.01, MK-4: *F*_(4,34)_ = 0.79, *p* = 0.54, total VK: *F*_(4,34)_ = 4.38, *p* < 0.01, MK-4/total VK: *F*_(4,34)_ = 2.99, *p* < 0.05]. Group differences within a given brain region were analyzed for individual vitamer by Student’s unpaired *t*-test. Statistically different from control group, ^∗^*p* < 0.05, ^†^*p* < 0.01.

In rats from the C group, MK-4 was by far the principal K vitamer in brain, representing ∼85% of total VK. In WVK rats, phylloquinone and total VK were significantly higher than in C rats (*p* < 0.05); however, MK-4 concentrations were 25–40% those of controls depending on the brain region (**Figure 5A**) and represented ∼20% of total VK (*p* < 0.01; **Figure 5B**). Furthermore, in C rats, MK-4 was unevenly distributed in the brain regions [*F*_(4,23)_ = 4.45; *p* < 0.01], higher concentrations being observed in prefrontal cortex, midbrain, and sensorimotor cortex. Interestingly, these regional differences totally disappeared in brains of WVK rats, concentrations of MK-4 being comparable across all brain regions [*F*_(4,34)_ = 0.79; *p* = 0.54; cf. figure legend for individual K vitamer statistics].

### Sphingolipid Status

**FIGURE 6:**
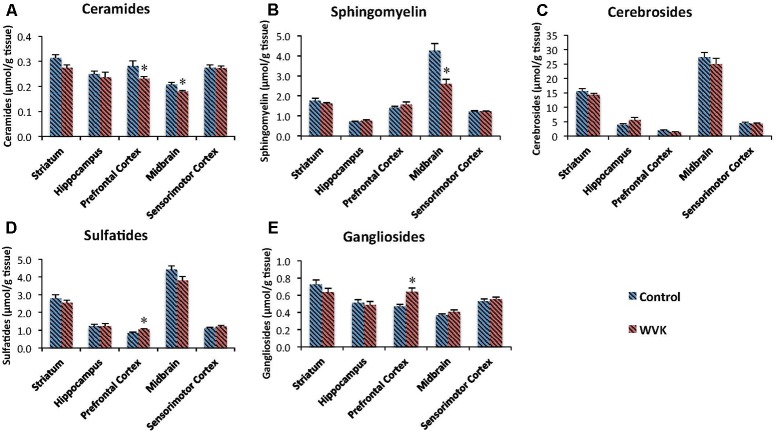
Effect of WVK treatment on sphingolipid profile in different brain regions in C and WVK groups. Figures show **(A)** ceramides; **(B)** sphingomyelin; **(C)** cerebrosides; **(D)** sulfatides; **(E)** gangliosides in striatum, hippocampus, prefrontal cortex, midbrain, and sensorimotor cortex. Values are mean ± SEM, *n* = 6–7 rats per group. Differences between brain regions were analyzed within C and WVK groups by one-way ANOVA, followed by Tuckey’s *post hoc* test. [Sphingomyelin C: *F*_(4,30)_ = 50.69, *p* < 0.0001; WVK: *F*_(4,29)_ = 8.76, *p* < 0.0001. Cerebrosides C: *F*_(4,30)_ = 149.3, *p* < 0.0001; WVK: *F*_(4,29)_ = 89.33, *p* < 0.0001. Sulfatides C: *F*_(4,30)_ = 107.8, *p* < 0.0001; WVK: *F*_(4,29)_ = 75.12, *p* < 0.0001. Ceramide C: *F*_(4,25)_ = 8.95, *p* < 0.0001; WVK: *F*_(4,24)_ = 5.19, *p* < 0.001. Gangliosides C: *F*_(4,30)_ = 13.8, *p* < 0.0001; WVK: *F_(_*_4,30)_ = 6.6, *p* < 0.05]. Group differences within a given brain region were analyzed by Student’s unpaired *t*-test. Statistically different from control group, ^∗^*p* < 0.05.

Concentrations of each class of sphingolipids generally varied across brain regions. In both groups of rats, highest concentration of sphingomyelin (C: *F*_(4,30)_ = 50.69, *p* < 0.0001; WVK: *F*_(4,29)_ = 8.76, *p* < 0.0001), cerebrosides (C: *F*_(4,30)_ = 149.3, *p* < 0.0001; WVK: *F*_(4,29)_ = 89.33, *p* < 0.0001), and sulfatides (C: *F*_(4,30)_ = 107.8, *p* < 0.0001; WVK: *F*_(4,29)_ = 75.12, *p* < 0.0001) were observed in the midbrain (*p* < 0.05). By contrast, the midbrain contained the least amounts of ceramides whereas the other regions showed statistically comparable concentrations (C: *F*_(4,25)_ = 8.95, *p* < 0.0001; WVK: *F*_(4,24)_ = 5.19, *p* < 0.001). In C rats, gangliosides varied significantly across brain regions with the striatum differing from all other regions and midbrain differing from striatum, hippocampus, and sensorimotor cortex [*F*_(4,30)_ = 13.8, *p* < 0.0001]. By contrast, regional gangliosides differences were largely mitigated in the WVK group with only the midbrain differing from prefrontal cortex and striatum, the other regions being statistically similar [*F_(_*_4,30)_ = 6.6, *p* < 0.05]. Furthermore, the WVK treatment was associated with decreased concentrations of ceramides in prefrontal cortex (↓20%, *p* < 0.05) and midbrain (↓13%, *p* < 0.05), decreased sphingomyelin in midbrain (↓39%, *p* < 0.01), and increased concentrations of gangliosides (↑25%) and sulfatides (↑20%) in the prefrontal cortex (*p* < 0.05; **Figure 6**).

#### HPTLC Ganglioside Fractions

**FIGURE 7:**
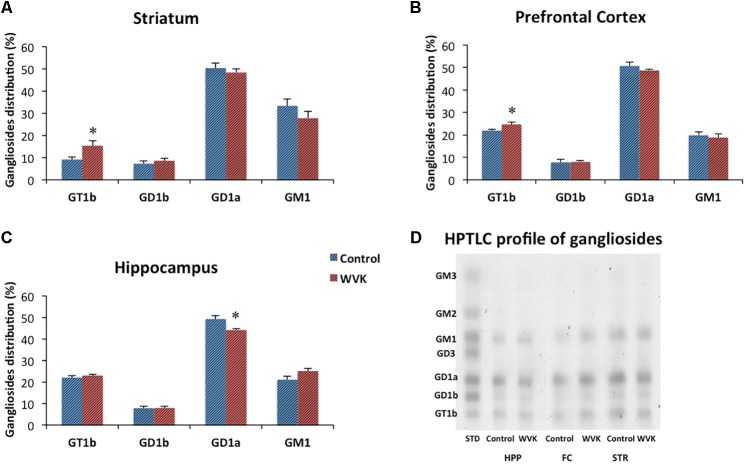
Effect of WVK treatment on ganglioside subtypes in specific brain regions. Figures show gangliosides in **(A)** striatum; **(B)** prefrontal cortex; **(C)** hippocampus; **(D)** ganglioside HPTLC gel. Values are mean ± SEM, *n* = 6–7 rats per group. Group differences within a given brain region were analyzed by Student’s unpaired *t*-test. Statistically different from control group, ^∗^*p* < 0.05.

In both C and WVK groups, gangliosides subtypes GD1b, GD1a, GT1b, and GM1 were differentially distributed across brain regions. Specifically, GD1a was present in highest concentrations in prefrontal cortex, striatum, and hippocampus followed by sensorimotor cortex (C: *F*_(4,30)_ = 87.36, *p* < 0.0001; WVK: *F*_(4,30)_ = 95.03, *p* < 0.0001) while significantly higher levels of GD1b (C: *F*_(4,30)_ = 15.77, *p* < 0.0001; WVK: *F*_(4,30)_ = 8.21, *p* < 0.0001) and GT1b (C: *F*_(4,30)_ = 60.77, *p* < 0.0001; WVK: *F*_(4,30)_ = 11.64, *p* < 0.0001) were observed in the midbrain. In the C group, GM1 was present in highest concentrations in the striatum and midbrain [*F*_(4,30)_ = 10.83, *p* < 0.0001], while in WVK rats, GM1 were highest in midbrain compared to other regions [*F*_(4,30)_ = 9.43, *p* < 0.0001; data not shown]. Importantly, WVK treatment resulted in a significant reduction in GD1a in the hippocampus (*p* < 0.05), and in significant increases in GT1b in the striatum and prefrontal cortex (*p* < 0.05; **Figure 7**).

### Relationship Between Brain MK-4 and Locomotion and Exploratory Behavior

**FIGURE 8:**
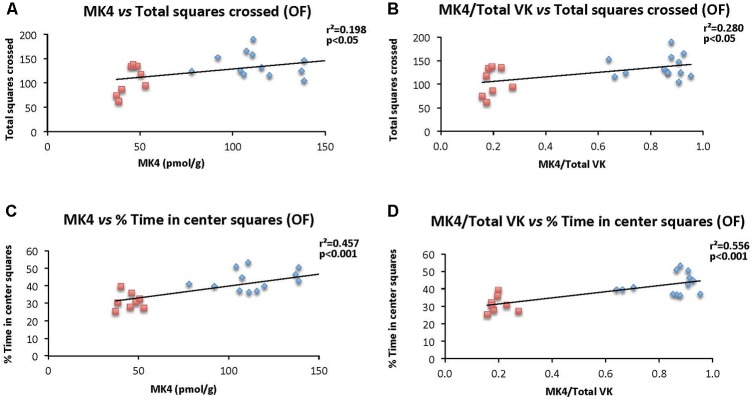
Relationship between brain MK-4 and locomotor activity and exploratory behavior. Figures show relationships of **(A)** MK-4 vs. total squares crossed; **(B)** MK-4/total VK vs. total squares crossed; **(C)** MK-4 vs. % time in central squares (OF); **(D)** MK-4/total VK vs. % time in central squares (OF). Data were analyzed by Pearson correlation test, *n* = 14–21 rats per correlation. Statistically significant, *p* < 0.05.

The specific relationships between brain MK-4 and locomotor activity and exploratory behavior as assessed in the open field test were determined. Specifically, correlations were established considering individual brain MK-4 (and MK4/total VK) and total squares crossed (**Figures 8A,B**), and % time in center squares (**Figures 8C,D**). As illustrated in the figures, both brain MK-4 and ratio MK4/total VK were strongly correlated with behavior, higher brain MK-4 and MK4/total VK ratios being associated with greater locomotor activity and exploratory behavior. In all cases, the poorer performances were observed in the WVK group. Additional positive correlations were also observed for locomotor activity (MK4/total VK vs. distance moved, *r*^2^ = 0.216, *p* < 0.05) and exploratory behavior (MK4 vs. center crossings, *r*^2^ = 0.263, *p* < 0.05; MK4/total VK vs. center crossings, *r*^2^ = 0.393, *p* < 0.01), rats from the WVK group performing again, less well.

## Discussion

In the present study, we provide new evidence of the detrimental effect of low VK status in brain on spatial learning ability, locomotor activity, and exploratory behavior, a phenotype associated with decreased cerebral MK-4 concentrations and altered sphingolipid pattern in key brain regions, notably of the ganglioside subtypes.

### Cognitive and Behavior Functions

When subjected to the Morris water maze test, rats from the WVK group showed higher latencies on day 2 which suggests a slower rate of spatial learning acquisition (**Figure 3A**). This result was neither confounded by motor dysfunction in the pool nor by differences in visual acuity as swim speed and performances on cue test were not different between groups. A similar finding of slower learning acquisition was also observed in aged animals (20 months), rats having been fed a low phylloquinone diet throughout their lives showing longer latencies on days 2 and 5 on the Morris water maze test (Carrié et al., 2011). Noteworthy, in this study, the low VK diet had no impact on cognition at 6 and 12 months suggesting that it was the chronic exposure to the low phylloquinone diet that lead to the detrimental effect on cognition. Interestingly, in a recent report by our group involving cognitively healthy older individuals who were administered an episodic memory task, those with lower VK status (sub-optimal status but not deficient) needed more exposure time to the words to be memorized than participants with higher VK status (Presse et al., 2013). Taken together and acknowledging that the impact of VK on cognition is mild and subtle, results from this and our previous studies advocate for a specific role for VK in the memory consolidation process.

In the present study, the WVK treatment was also associated with lower locomotor activity and exploratory behavior (**Figure 4**). These results are in agreement with those from an older study in which short-term warfarin treatment was associated with a significant shift from more exploratory to less exploratory behavior in male Sprague-Dawley rats (Cocchetto et al., 1985). By contrast, locomotion and exploratory behavior were not affected in our aged rats who had consumed a low phylloquinone diet since weaning (Carrié et al., 2011). This discrepancy could be due to various factors including different experimental design, i.e., pharmacological vs. nutritional approach. Another factor could be the sex of the animals. In the present study, male rats were investigated, whereas our aging study was conducted in female rats who are known to be more resistant to VK deficiency (Metta et al., 1959; Gustafsson et al., 1962; Ferland et al., 2016a).

### VK Status

Strong evidence has been provided in recent years supporting the bioconversion of phylloquinone into MK-4, a reaction catalyzed by the UbiA prenyltransferase domain-containing 1 enzyme (Nakagawa et al., 2010) which involves the menadione form of VK as an intermediate (Hirota et al., 2013). In the present study, MK-4 was by far the principal K vitamer in brains of C rats, representing ∼85% of total VK (**Figure 5**), a result in line with previous reports (Thijssen and Drittij-Reijnders, 1994; Thijssen et al., 1996; Carrié et al., 2011). By contrast, MK-4 concentrations in brains of WVK rats represented no more than 20% of total VK. In a study by Thijssen et al. (1996) administration of 1 mg/kg (s.c.) of warfarin to rats fed standard amounts of phylloquinone and/or menadione was associated with MK-4 concentrations that were ∼20% those of control animals. In an older report, significant decreases in MK-4 concentrations were observed in kidneys and hearts of rats treated with the coumarin drug, Dicoumarol (Taggart and Matschiner, 1969). The fact that, in the present study, brain MK-4 concentrations remained low in WVK rats despite an excess of phylloquinone in brain suggests an alteration of the MK-4 biosynthetic pathway in the presence of warfarin. The partial loss of MK-4 brain regional differences in the WVK group also supports this notion. Future studies should include assessment of the UbiA prenyltransferase domain-containing 1 enzyme to determine whether its activity is inhibited by warfarin *in vivo*.

The highly increased phylloquinone concentration in brains of WVK rats suggests that phylloquinone can reach this organ. Previous studies from our groups (Carrié et al., 2004; Ferland et al., 2016a) and others (Thijssen and Drittij-Reijnders, 1994; Thijssen et al., 1996) have shown phylloquinone concentrations to increase in brain with phylloquinone intake, and intravenous supplementation (Ronden et al., 1998). To date, details of the phylloquinone transport into CSF and brain are not known. Unlike other fat-soluble vitamins, VK has no known carrier protein. In the circulation, phylloquinone is principally carried in triacylglycerol-rich lipoproteins (>50%) with each of the LDL and HDL fractions accounting for ∼15% of the circulating vitamer (Lamon-Fava et al., 1998). Noteworthy, the scavenger receptor class B type 1 has been shown to promote the uptake of HDL-associated vitamin E (i.e., a-tocopherol) in porcine brain capillary endothelial cells, suggesting this receptor could, in part, be responsible for the vitamin E transport across the blood brain barrier (Goti et al., 2001). Alpha-tocopherol and phylloquinone have similar molecular weights and are both associated with the lipoprotein fractions (Kayden and Traber, 1993; Lamon-Fava et al., 1998). Whether phylloquinone enters the brain through a route similar to that of a-tocopherol remains to be determined.

Finally, it should be emphasized that brain MK-4 was found to be strongly correlated with behavior, higher concentrations being associated with increased locomotor activity and exploratory behavior (**Figure 8**). Such correlations have not, to our knowledge, been reported before, and provide additional support for the modulatory role of MK-4 in behavior.

### Sphingolipid Analyses

Sphingolipids are pivotal constituents of the plasma membranes and are important for proper brain functions. In the present study, sphingolipid profile was altered as a function of warfarin treatment and depletion of brain MK-4. While gangliosides varied significantly across brain regions with the striatum containing the highest amounts, regional differences were largely mitigated in the WVK group. Sphingolipid profiles similar to those in the C group were previously observed in 6- and 20-month-old Sprague-Dawley rats who had been fed various levels of phylloquinone since weaning (Carrié et al., 2004, 2011). Furthermore, WVK treatment modified the sphingolipid content of the midbrain and prefrontal cortex. Specifically, sphingomyelin was found to be particularly decreased in the midbrain whereas gangliosides were increased in the prefrontal cortex. Ceramides and sulfatides were, respectively, decreased and increased in the prefrontal cortex. Sulfatides and cerebrosides are involved in the process of oligodendrocyte differentiation (Jana et al., 2009) and contribute to the maintenance of myelin and axon structures of the central nervous system (Schmitt et al., 2015). Ceramides, which can be generated from *de novo* synthesis or from hydrolysis of sphingomyelin through the action of sphingomyelinases (**Figure 1**), are intracellular messenger with potent cell signaling actions. Studies conducted *in vitro* and *in vivo* suggest important roles for sphingomyelin-ceramide signaling in the regulation of cell proliferation, differentiation and survival (Cutler and Mattson, 2001; Ledesma et al., 2012). In a series of studies, the acid sphingomyelinase–ceramide pathway was shown to play an important role in genetically induced depression (Kornhuber et al., 2014; Müller et al., 2017), mice over-expressing acid sphingomyelinase activity and increased ceramide concentrations showing decreased neurogenesis, neuronal maturation/survival and depression-like behavior (Gulbins et al., 2013). Evidence was also reported for a role of ceramide in stress-induced depression, various unavoidable stressors being associated with enhanced acid sphingomyelinase activity and/or ceramide levels in the brain (Gulbins et al., 2013; Oliveira et al., 2016). The fact that animals from the WVK group were not found to be more anxious than controls on the elevated plus maze test are in line with what appears to be a downregulation of the sphingomyelin–ceramide pathway in this WVK model.

Gangliosides are major components of cell membranes where they participate in key neuronal functions such as axon outgrowth/regeneration, nerve cell excitability, and myelin stability (Schengrund, 2015; Schnaar, 2016). In the present study, WVK treatment resulted in a significant reduction in GD1a in the hippocampus (*p* < 0.05), and in significant increases in GT1b in the striatum and frontal cortex (*p* < 0.05; **Figure 7**). In addition to their general functions, ganglioside subtypes have been involved in specific conditions and physiological processes. Ganglioside GD1a has notably been shown to be reduced in frontal gray matter and white matter in patients with Rett syndrome, a neurodevelopmental disorder associated with autism-like behavior and behavioral disturbances (Lekman et al., 1991). In a genetic mouse model of the disease, GD1a concentrations were found to be decreased by 15% in the cerebrum and brainstem (Seyfried et al., 2009). Ganglioside GD1a has also been involved in anti-inflammatory processes (Wang et al., 2008; Nikolaeva et al., 2015). In a recent study, GD1a inhibited *Escherichia coli* lipopolysaccharide-induced inflammation in murine RAW264.7 macrophages by suppressing phosphorylation of mitogen-activating protein kinases and nuclear translocation of NF-κB family members through the Toll-like receptor 4 signaling pathway (Wang et al., 2015). The specific neuroprotective role of GD1a was further evidenced in the study by Bernardo et al. (2009) where increased brain levels of GD1a, through inhibition of GD3S and synthesis of gangliosides from the b-series, was shown to mitigate Aβ-associated neurotoxicity and rescue spatial-memory impairment in the double-transgenic (APP/PSEN1) mouse model of Alzheimer’s disease. By contrast, ganglioside GT1b has been associated with neurotoxicity and pro-inflammatory conditions. In a series of experiments, GT1b was shown to activate microglia and induce the production of inflammatory mediators such as IL-1β, iNOS, TNF-α, and COX-2 (Pyo et al., 1999). Microglial activation was subsequently shown to be mediated by protein kinase C and NADPH oxidase (Min et al., 2004). Ganglioside GT1b has also been found to be neurotoxic to dopaminergic neurons *in vitro* (Chung et al., 2001) through a modulation of the Akt/GSK-3/Tau signaling pathway (Chung et al., 2010). *In vivo*, injection of GT1b into the substantia nigra resulted in the death of nigral neurons, a finding associated with activation of microglia (Ryu et al., 2002).

In light of their biological actions, the observed results for GT1b in the striatum, frontal cortex, and hippocampus could have contributed to the poor performance of the WVK-treated rats in the Morris water maze and open field as these regions are directly linked to the tasks associated with these tests. Although it is well established that hippocampus supports the spatial processing demands of the water maze (D’Hooge and De Deyn, 2001), studies have recently highlighted significant implications for striatum and prefrontal cortex in the Morris water maze paradigm (Woolley et al., 2013). As for striatum, it has long been known to play important roles in motor behavior and locomotor activity, two components associated with the open field test (David et al., 2005; Dang et al., 2006).

Limitations of this study include the fact that except for the ganglioside family, total measures of sphingolipids were obtained, possibly missing on the implications of specific sphingolipids sub-species. When investigated in the context of behavioral extinction, only specific ceramide species were found to be affected (Huston et al., 2016). Another limitation of the study concerns the fact that activity of the enzymes involved in sphingolipid metabolism were not assessed. In future studies, sphingolipid sub-species should be quantitated using mass spectrometry technology and their respective metabolic enzymes be determined. Finally, animals from the WVK group had lower food intake than controls suggesting that results could have been influenced by overall lower intake of energy, and/or specific nutrients other than VK.

The present study aimed to gain mechanistic insight on the impact of targeted brain VK depletion on cognition as it relates to sphingolipid metabolism outside the context of aging. This was achieved through the concurrent administration of large amounts of warfarin to deplete the brain and large doses of phylloquinone to maintain coagulation. The amount of warfarin involved in this experimental paradigm does not compare in any way to the doses used in the clinical setting where VK antagonists such as warfarin are prescribed for the prevention of thromboembolic diseases; the primary end-point of VK antagonists being to suppress the coagulation cascade (Ansell et al., 2008). In recent years, our group has conducted a number of studies in elderly patients undergoing anticoagulation therapy with VK antagonists and while use of these drugs was found to be independently associated with lower cognitive function in cross-sectional analyses, results did not suggest cumulative long-term detrimental effects (Annweiler et al., 2015; Ferland et al., 2016b). Hence, whether VK antagonists alter brain function through their impact on sphingolipid metabolism or other mechanisms, or benefit cognition through their antithrombotic effects, remains a topic of current investigation.

## Conclusion

In conclusion, this study provides further evidence that targeted depletion of MK-4 in brain is associated with cognitive impairment, lower locomotor activity, and exploratory behavior, and with an alteration of sphingolipids in key brain regions, notably those of the ganglioside family. Results also suggest that, *in vivo*, phylloquinone in brain is not bio-transformed into MK-4 in the presence of W. Future studies should focus on the risk–benefit balance of VKA treatment as it relates to cognition and behavior. Finally, results from this study confirm the role of VK in brain and underline the importance of commonly consumed diets in providing this nutrient in adequate amounts.

## Author Contributions

GF designed the study. ST-N performed the experiments. BO and JR supervised the work. GF and ST-N analyzed the data and wrote the manuscript.

## Conflict of Interest Statement

The authors declare that the research was conducted in the absence of any commercial or financial relationships that could be construed as a potential conflict of interest.

## Abbreviations

C: control group
HPTLC: high-performance thin-layer chromatography
MK-4: menaquinone-4
VK: vitamin K
WVK: warfarin plus phylloquinone treatment
